# Transactions of the Association of American Physicians

**Published:** 1889-03

**Authors:** 


**Transactions of the Association of American Physicians.** Volume III. Printed for the Association. Philadelphia. 1888.

This is a creditable volume, and contains many important papers. Typhoid Fever receives considerable attention. It is considered as to its geographical distribution and its relation to fevers of malarial origin, by W. W. Johnston, M.D., of Washington; as to the management of the stage of convalescence, by James H. Hutchinson, M. D., of Philadelphia; as to some forms of paralysis following the fever, by George Ross, M. D., of Montreal.

Certain diseases of the heart are the next to claim attention. These are: "Fatty Overgrowth," by F. Forchheimer, M. D., of Cincinnati; "Disturbances of its Rhythm with Reference to their Causation and Value in Diagnosis," by G. Baumgarten,

M. D., of St. Louis; "Cardiac Changes in Chronic Bright's Disease," by A. L. Loomis, M. D., of New York; and a physiological paper on "The Pulse-wave Velocity and Ventricular Close-time in Health," by James K. Thacher, M. D., of New Haven. Dr. Frank Donaldson, of Baltimore, considers "The Diagnostic Significance of the Mitral Presystolic Murmur," and Dr. George L. Peabody reports a case of a "Pin Imbedded in a Woman's Heart."

Besides the paper of Dr. Loomis above referred to, papers relating to diseases of the kidneys are presented as follows: Dr. Robert T. Edes, of Washington, and Dr. Edward G. Janeway, of New York, discuss "The Value of Albumin and Casts, and of Venal Inadequacy in the Diagnosis of Kidney Disease;" and Dr. James Tyson, of Philadelphia, gives a paper on "The Relation of Albuminuria to Life Assurance." These three groups of papers make up the bulk of the volume. The other papers are: "Causal Therapeutics in the Infectious diseases," by Dr. J. C. Wilson, of Philadelphia; "The Relation between Trophic Lesions and Diseases of the Nervous System," by Dr. E. C. Seguin, of New York; "Effects of Spinal Concussion on the Reflexes," by Dr. E. T. Miles, of Baltimore; "Contributions to the Anatomy and Pathology of the Thymus Gland," by Dr. A. Jacobi, of New York; "Recent Researches Relating to the Etiology of Yellow Fever," by Dr. George M. Sternberg; "Pulsating Pleurisy," by Dr. Wm. Osler, of Philadelphia; "Antiseptic Medication," by Dr. F. P. Henry, of Philadelphia; "Phthisis Pulmonalis, the Basis of its Therapeutics," by Dr. S. D. Solly, of Colorado; "Creosote as a Remedy in Phthisis Pulmonalis," by Dr. Beverly Robinson, of New York; and "The Continued Fevers of the Mississippi Valley," by Dr. P. G. Robinson, of St. Louis.

